# Metabolic and Tissue-Specific Regulation of Acyl-CoA Metabolism

**DOI:** 10.1371/journal.pone.0116587

**Published:** 2015-03-11

**Authors:** Jessica M. Ellis, Caitlyn E. Bowman, Michael J. Wolfgang

**Affiliations:** Department of Biological Chemistry, Johns Hopkins University School of Medicine, Center for Metabolism and Obesity Research, Baltimore, Maryland 21205 United States of America; Monash University, AUSTRALIA

## Abstract

Acyl-CoA formation initiates cellular fatty acid metabolism. Acyl-CoAs are generated by the ligation of a fatty acid to Coenzyme A mediated by a large family of acyl-CoA synthetases (ACS). Conversely, acyl-CoAs can be hydrolyzed by a family of acyl-CoA thioesterases (ACOT). Here, we have determined the transcriptional regulation of all ACS and ACOT enzymes across tissues and in response to metabolic perturbations. We find patterns of coordinated regulation within and between these gene families as well as distinct regulation occurring in a tissue- and physiologically-dependent manner. Due to observed changes in long-chain ACOT mRNA and protein abundance in liver and adipose tissue, we determined the consequence of increasing cytosolic long-chain thioesterase activity on fatty acid metabolism in these tissues by generating transgenic mice overexpressing a hyperactive mutant of Acot7 in the liver or adipose tissue. Doubling cytosolic acyl-CoA thioesterase activity failed to protect mice from diet-induced obesity, fatty liver or insulin resistance, however, overexpression of Acot7 in adipocytes rendered mice cold intolerant. Together, these data suggest distinct modes of regulation of the ACS and ACOT enzymes and that these enzymes act in a coordinated fashion to control fatty acid metabolism in a tissue-dependent manner.

## Introduction

Free fatty acids must be converted to fatty acid-Coenzyme A, or acyl-CoAs, in order to be shuttled into nearly all fatty acid metabolic processes including protein acylation, membrane phospholipid biosynthesis, energy storage, oxidation for energy production, and the synthesis of signaling lipids. Excessive accumulation of intracellular acyl-CoAs can be toxic, promote mitochondrial stress and increase the production of damaging free radicals [1,2]. Therefore, dysregulated lipid metabolism, originating with the formation of acyl-CoAs, is a cornerstone of multiple diseases including obesity and diabetes. Acyl-CoA formation and degradation is mediated by two enzyme families. Acyl-CoA synthetases (ACS) generate acyl-CoA from a free fatty acid (FA) and CoA in an ATP-dependent manner, and acyl-CoA thioesterases (ACOT) hydrolyze acyl-CoAs to a free FA and CoA. In the mouse, there are 25 ACS and 15 ACOT enzymes which differ in tissue distribution, subcellular location, and substrate specificity [3–7]. Formation of a membrane-impermeable acyl-CoA traps the fatty acid in the cellular compartment where it was synthesized and is obligate for the acyl-transfer reactions that direct its subsequent metabolism, whereas the hydrolysis of the acyl-CoA to a free fatty acid both deactivates the fatty acid and allows its exit from the cell or organelle [8]. Because acyl-CoA formation is an initial step in fatty acid metabolism, it has been proposed that the activating and deactivating enzymes, i.e. ACS and ACOTs, act to direct the metabolic fate of the fatty acid by channeling the substrate towards or away from downstream enzymes [9]. The ACS and ACOT enzymes may also regulate fatty acid metabolism by controlling cellular concentrations and compartmentalization of free fatty acid, acyl-CoA, free CoA, and AMP/ATP [10–13].

Several of these enzymes have been shown to play unique roles in regulating fatty acid metabolism in cell culture models and in a tissue-dependent manner *in vivo* [14,15]. However, the physiological roles of the majority of ACS and ACOT enzymes have yet to be identified. In addition, it remains unknown how these enzyme families act in a concerted manner to regulate fatty acid metabolism, a mechanism of regulation predicted in part due to the convergence of substrate specificity, subcellular localization, and multimerization of these enzymes. Here, we have determined the transcriptional regulation of all ACS and ACOT enzymes across a broad range of tissues and under multiple physiological metabolic perturbations. We show a diverse pattern of expression and transcriptional regulation of these genes highlighting the importance of tissue-specific regulation of acyl-CoA metabolism. These data support the notion of distinct roles and regulatory mechanisms for these enzymes and suggest that acyl-CoA synthetases and thioesterases act in a coordinated fashion to regulate fatty acid metabolism.

To determine the functional role of cytosolic acyl-CoA thioesterase regulation of fatty acid metabolic flux *in vivo*, we generated a transgenic mouse model that expresses a hyperactive cytosolic long-chain acyl-CoA thioesterase, Acot7, in a tissue-specific manner. Surprisingly, increasing cytosolic thioesterase activity in the liver or adipose tissue did not protect the mice from fasting- or diet-induced fatty liver, obesity, or insulin resistance. Increasing thioesterase activity in adipose tissue, however, did impair cold-induced thermogenesis. These data suggest complex and coordinated control of fatty acid metabolic flux through the concerted activities of both acyl-CoA thioesterases and synthetases.

## Results

### Metabolic and tissue dependent transcriptional regulation of Acyl-CoA Thioesterase and Synthetase Enzymes

**Fig 1:**
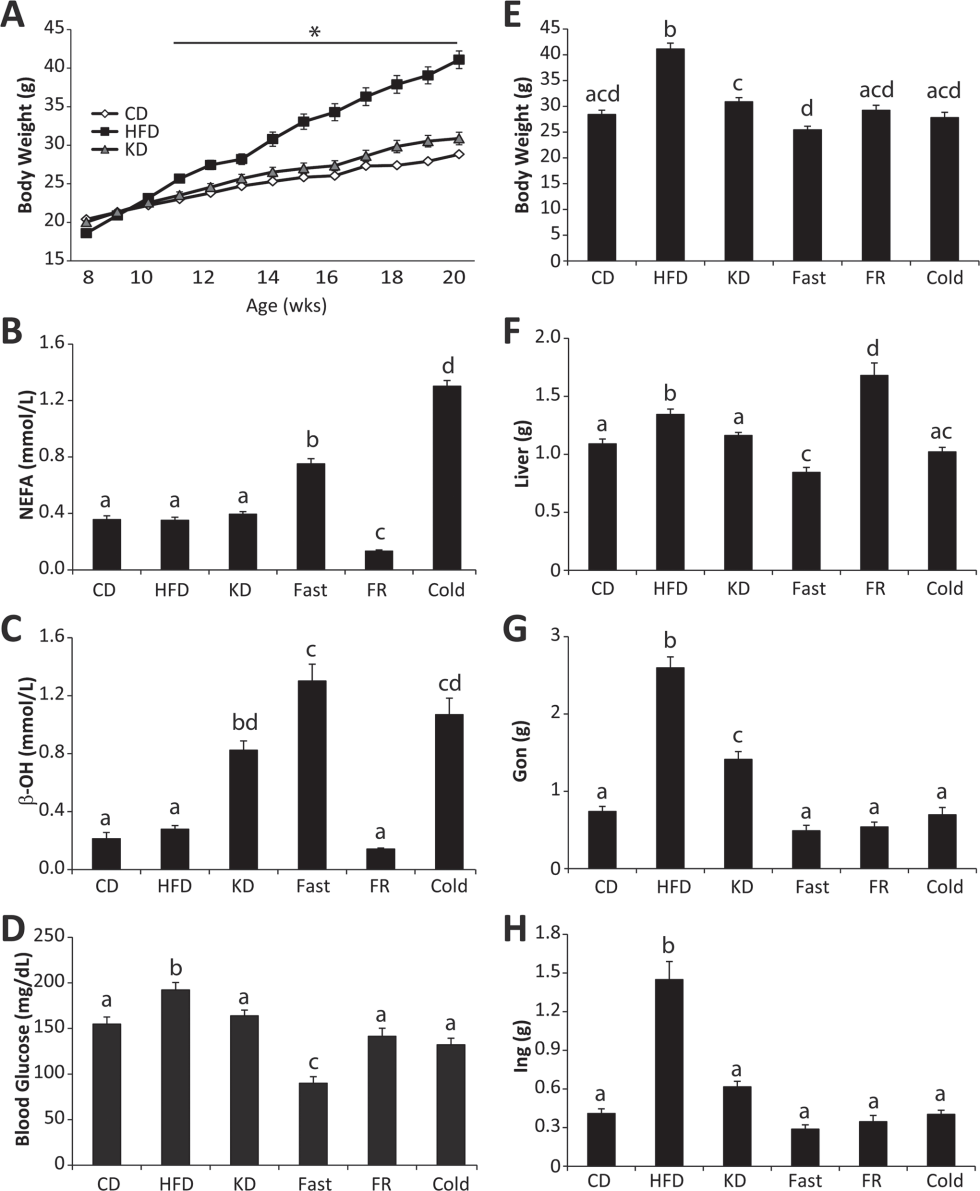
Nutritional perturbation in mice. **(A**) Body weights of male C57Bl/6J mice for 12 weeks on either control diet (CD), high-fat diet (HFD), or ketogenic diet (KD) n = 15–30. (**B**) Serum non-esterified free fatty acids (NEFA), (**C**) beta-hydroxybutyrate (β-OH), and (**D**) blood glucose in CD, HFD, KD, Fasted (Fast), Fasting-Refed (FR), and cold exposed mice, n = 8–10. **(E**) Whole body, **(F**) liver, (**G**) gonadal white adipose, and (**H**) inguinal white adipose weight in CD, HFD, KD, Fast, FR, and cold exposed mice, n = 10–15. Data represent mean ± SEM, * represents p≤0.05 by Student’s t-test relative to the CD group. Significant differences among group means are represented by letters and were determined by Tukey multiple comparison tests (p<0.05) after one-way ANOVA.

To better understand the metabolic regulation of the initial steps in cellular fatty acid metabolism, the ligation to Coenzyme A, we profiled the transcriptional response of enzymes known to activate (acyl-CoA synthetases) or deactivate (acyl-CoA thioesterases) fatty acids in response to nutritional perturbation *in vivo*. We chose six metabolic conditions: 1) control diet (CD) fed, 2) high-fat (60%) diet (HFD) fed, 3) ketogenic diet (KD) fed, 4) CD fed mice fasted overnight (Fast), 5) CD fed mice fasted for 14 hours then refed for 12 hours overnight (FR), and 6) CD fed mice exposed to 4°C for 4 hours prior to tissue harvest (Cold) (see diet composition **S1 Table**). Male C57Bl/6J mice were randomly placed on these diets for 12 weeks. These dietary conditions were chosen because they elicit profound and distinct effects on whole body fatty acid metabolism. As expected the HFD, relative to the CD fed mice, led to an increase in body weight, fatty liver, increased adiposity and elevated blood glucose (**Fig. 1A-H**). The KD did not increase body weight, but increased adiposity and caused hyperketonemia (**Fig. 1A-H**). Overnight fasting resulted in hyperketonemia, elevated serum free fatty acids, lowered blood glucose, and a loss of 11% body weight (**Fig. 1B-H**). Fasting-refeeding reversed many of the effects caused by fasting, with the exception that refeeding led to increased liver weight and reduced adiposity relative to the control diet fed mice (**Fig. 1B-H**). As expected, cold exposure caused a lowering of blood glucose, hyperketonemia, and increased serum free fatty acids (**Fig. 1B-H**). These nutritional perturbations show robust differences in adiposity and circulating components of fatty acid metabolism.

To better understand how these gatekeeper enzymes that control fatty acid entry into cellular metabolic pathways are regulated under these circumstances, we profiled the transcriptional regulation of the 15 fatty acid deactivating acyl-CoA thioesterase enzymes and 25 fatty acid activating acyl-CoA synthetases encoded in the mouse genome across tissues exposed to these dietary conditions (See **S2 Table** for gene abbreviations). The tissues profiled were liver, kidney, heart (left ventricle), gonadal white adipose tissue (Gon/gWAT), inguinal white adipose tissue (Ing/iWAT), soleus (red) muscle, plantaris (white) muscle, duodenum (gut) and whole brain. We also profiled brown adipose tissue (BAT) from the control diet fed mice and mice exposed to 4°C for four hours. Therefore, 10 tissues were collected from 6 disparate nutritional perturbations chosen for their unique requirements and roles in regulating whole animal fatty acid homeostasis.

### Thioesterase and synthetase regulation by metabolic perturbation

**Fig 2:**
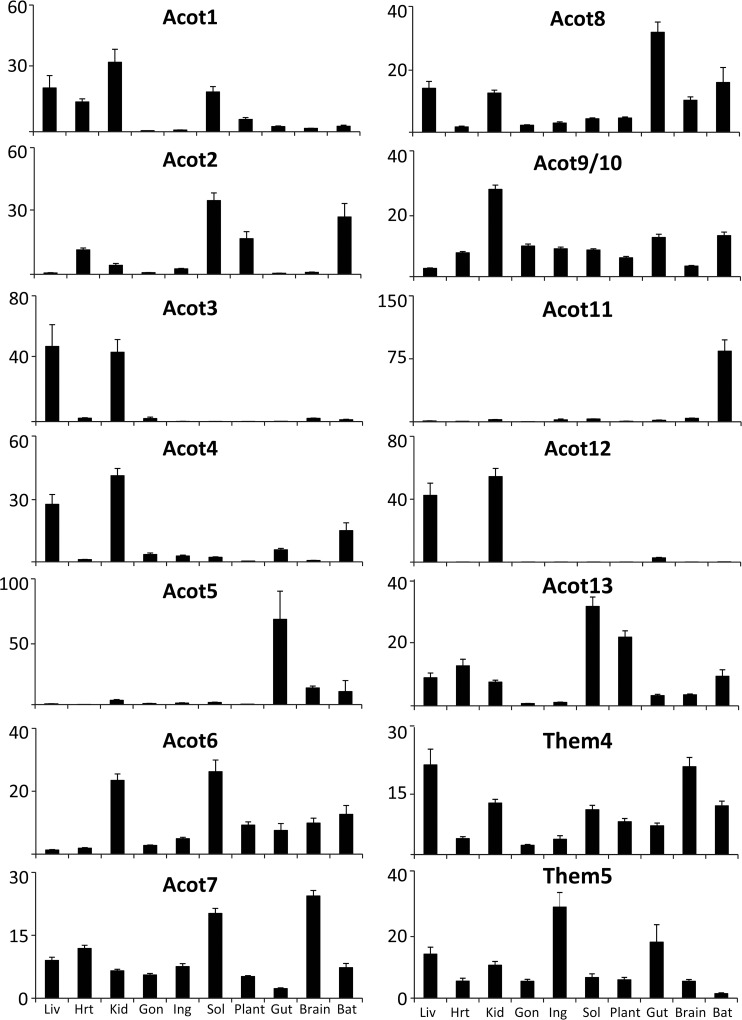
Relative tissue distribution of the acyl-CoA thioesterases. Percent tissue distribution of each enzyme was determined by qPCR in relation to the sum of its expression across all tissues and all dietary conditions for the liver (Liv), heart (Hrt), kidney (Kid), gonadal white adipose (Gon), inguinal white adipose (Ing), soleus muscle (Sol), plantaris muscle (Plant), duodenum (Gut), whole brain (Brain), and brown adipose tissue (BAT), n = 20–45. Data represent mean percent ± SEM.

**Fig 3:**
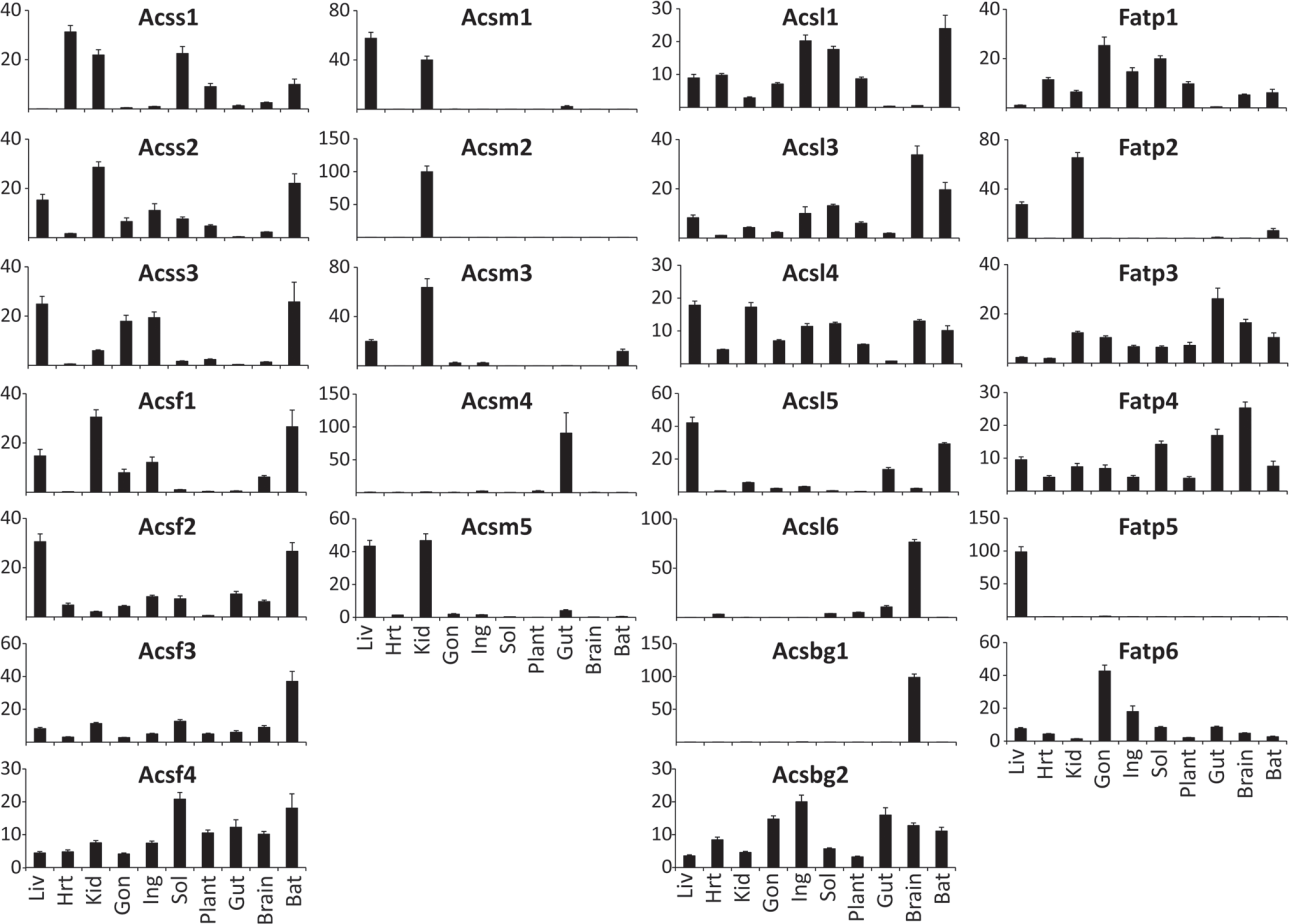
Relative tissue distribution of the acyl-CoA synthetases. Percent distribution of each enzyme was determined by qPCR in relation to the sum of its expression across all conditions for the liver (Liv), heart (Hrt), kidney (Kid), gonadal white adipose (Gon), inguinal white adipose (Ing), soleus muscle (Sol), plantaris muscle (Plant), duodenum (Gut), whole brain (Brain), and brown adipose tissue (BAT), n = 20–45. Data represent mean percent ± SEM.

To understand how these metabolic perturbations altered the enzymes that initially generate or hydrolyze acyl-CoAs, we profiled the mRNA abundance of the 15 identified mouse acyl-CoA thioesterases and 25 acyl-CoA synthetases by real time qPCR in the tissues and dietary manipulations described above. We could not distinguish between *Acot9* and *Acot10* due to a ~97% mRNA sequence identity, therefore this data is expressed as *Acot9/10*. Each of the ACOT and ACS enzymes is known to have distinct tissue-specific expression patterns [16–17]. Here, we represent the cross-tissue expression for each enzyme as the percentage relative to the sum of expression across all tissues. We find very diverse expression across tissues with some enzymes being expressed widely, such as *Them4*, *Them5*, *Acsl4*, and *Acsf4*; whereas other enzymes are highly expressed in one or two tissues, such as *Acot3*, *Acot5*, *Acot11*, *Acot12*, *Acsm1*, *Acsm2*, *Acsm4*, *Acsl6*, *Fatp5*, and *Acsbg1* (**Fig. 2 and Fig. 3**). A majority of the ACS and ACOT enzymes are enriched in one or a few tissues as opposed to being expressed widely, and we postulate that this reflects the evolutionary diversification of these enzyme families in order to regulate acyl-CoA metabolism in a tissue-specific manner.

**Fig 4:**
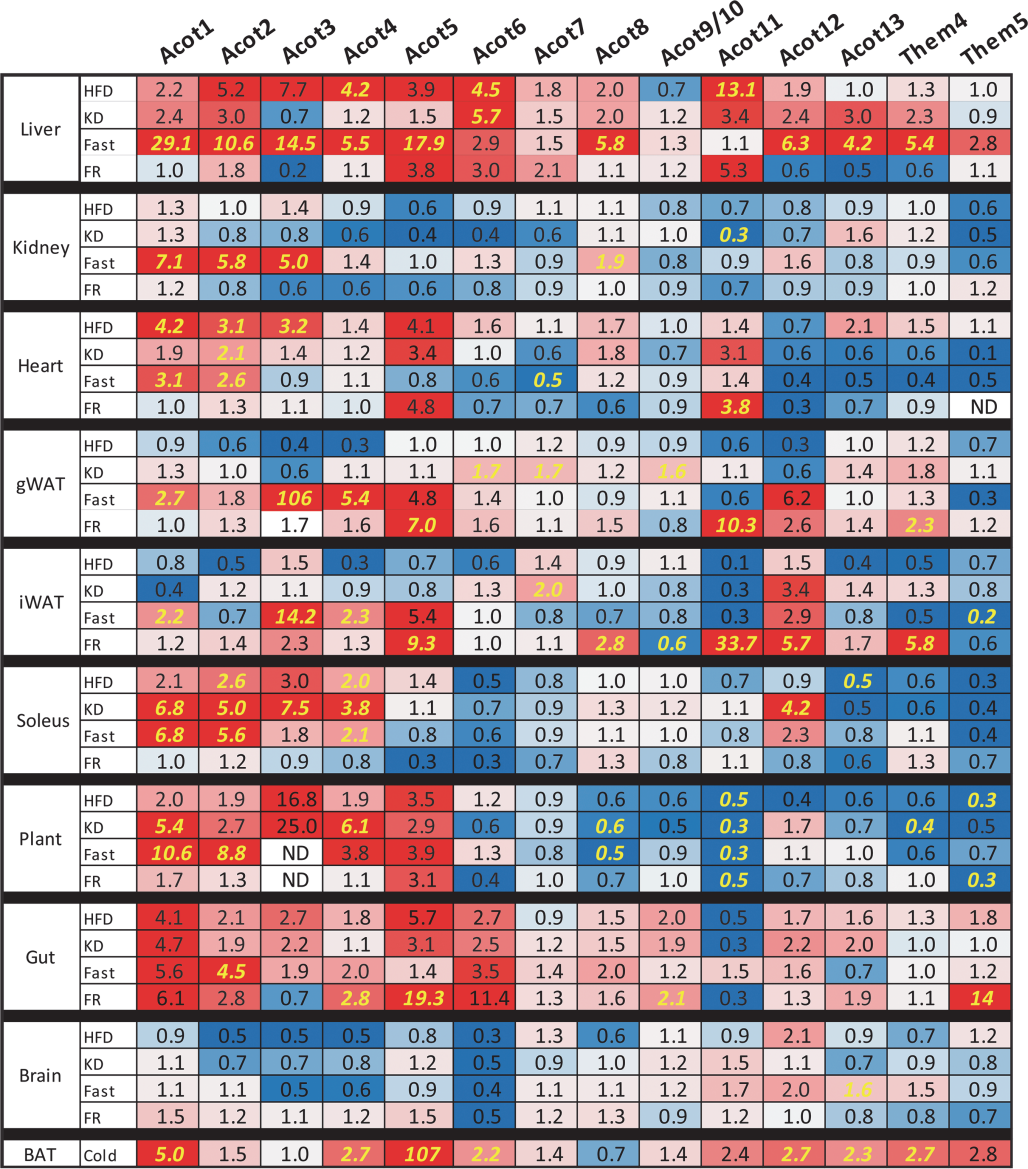
Nutritional modulation of ACOT enzymes. Fold-change of tissue mRNA abundance for each gene, relative to control diet, for high-fat diet (HFD), ketogenic diet (KD), overnight fasted (Fast), overnight fasted followed by 12-hour refeeding (FR), or cold exposed (Cold) mice (n = 6–8). ND indicates not detectable. Significant differences between CD and all other groups represented in *yellow* and were determined by Tukey multiple comparison tests (p<0.05) after one-way ANOVA, except for cold treatment which was analyzed by Student’s t-test. Complete statistical analysis via one-way ANOVA is provided in **S1 Fig.**

**Fig 5:**
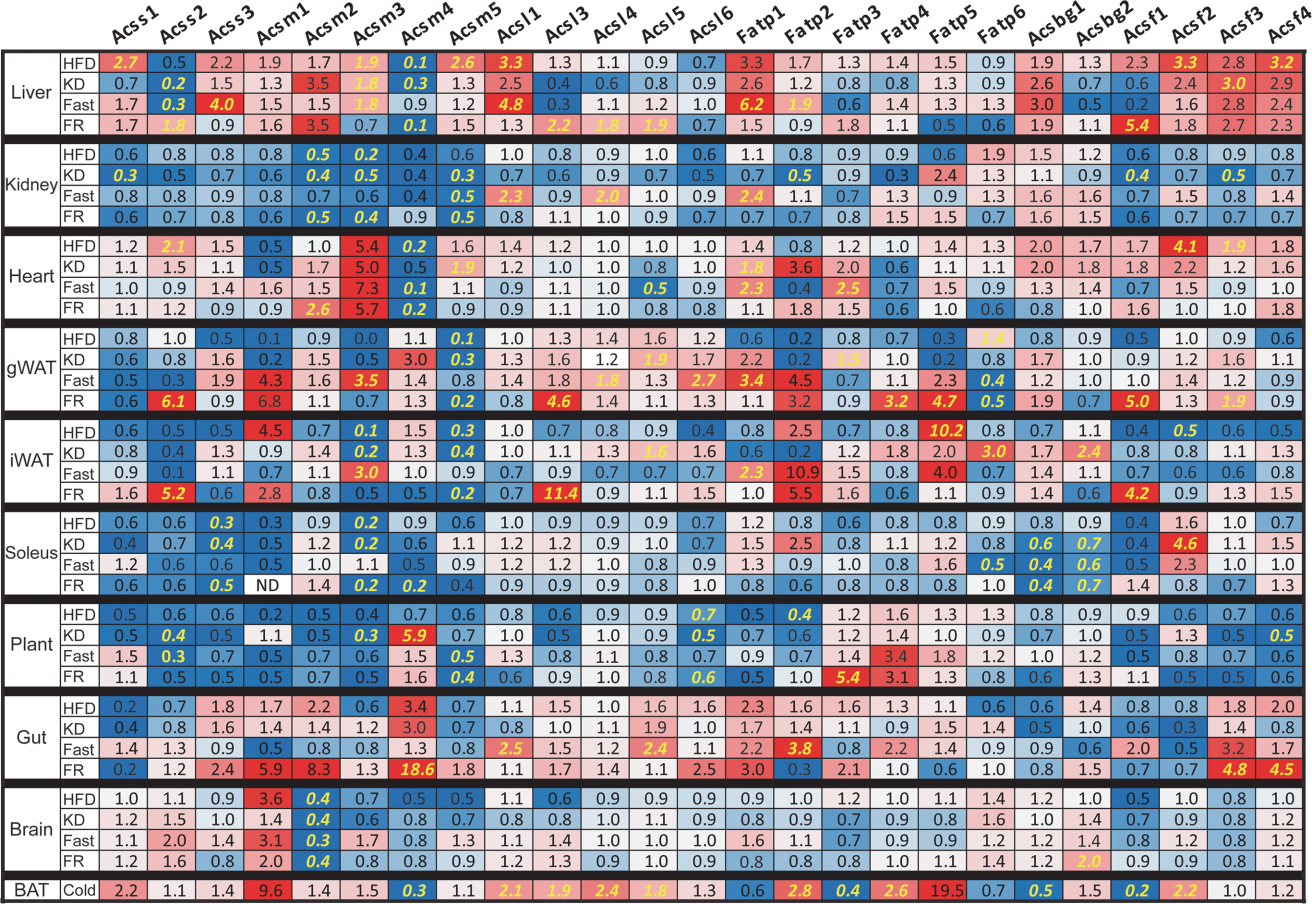
Nutritional modulation of ACS enzymes. Fold-change of tissue mRNA abundance for each gene, relative to control diet, for high-fat diet (HFD), ketogenic diet (KD), overnight fasted (Fast), overnight fasted followed by 12-hour refeeding (FR), or cold exposed (Cold) mice (n = 6–8). ND indicates not detectable. Significant differences between CD and all other groups represented in *yellow* and were determined by Tukey multiple comparison tests (p<0.05) after one-way ANOVA except for cold treatment which was analyzed by Student’s t-test. Complete statistical analysis via one-way ANOVA is provided in **S2 Fig.**

To understand how the metabolic conditions altered gene expression of the ACOT and ACS gene families, we compared how each gene was changed by condition and tissue relative to the control diet fed group; these data are expressed as fold-change relative to the control diet (**Fig. 4 and Fig. 5**). Significant differences for all pairwise comparisons are shown in **S1 Fig. and S2 Fig.** Each metabolic condition induced large variations in gene expression of the ACOT and ACS gene families across tissues. We made several interesting observations from this data, for example, the type one ACOTs (*Acot1*-*Acot6*) are up-regulated in fasted and HFD livers (**Fig. 4**). These highly homologous ACOTs are clustered on mouse chromosome 12 and human chromosome 14 and are reported to be coordinately regulated by PPARα [16]. However, the regulation of the type one ACOTs was less well-coordinated in tissues other than liver. For example, while none of the type one ACOTs were highly expressed in white adipose, *Acot3* was the most up-regulated of all ACOT enzymes in fasted gWAT and iWAT with a 106-fold and 14-fold increase, respectively. In the skeletal muscle, *Acot1*, *Acot2*, and *Acot6* were the predominant type I ACOTs however they were discordantly regulated with *Acot1* and *Acot2* up-regulated by fasting, KD, and HFD while *Acot6* remained unchanged by all conditions. *Acot5* was predominantly expressed in the gut and up-regulated by conditions of nutritional availability, namely HFD, KD, and FR, a surprising regulation considering the putative antagonistic nature of thioesterase activity against fatty acid metabolism.

Similar to *Acot1-6*, *Acsm1-5* are genetically clustered on mouse chromosome 7. However, unlike *Acot1-6*, the ACSM genes were not coordinately transcriptionally regulated (**Fig. 5**). *Acsm4* was most abundantly expressed in the gut and was up-regulated in response to increased nutrient availability. With the exception of *Acsm4*, the rest of the ACSM genes are enriched in the kidney and were down-regulated in the kidney by HFD, KD, and FR. This likely reflects the low abundance of medium-chain fatty acids present in the diets and the high availability of long-chain fatty acids under these dietary conditions. Conversely, *Acsm1*, *Acsm3*, and *Acsm5* were up-regulated in HFD liver, despite low dietary abundance of medium-chain fatty acids. The ACSM enzymes may activate fatty acid metabolic intermediates derived from the metabolism of longer chain fatty acids, thereby increasing the efficiency of the liver in lipid storage and repackaging of lipoprotein particles.

Both *Acss2*, which activates cytosolic acetate for *de novo* lipogenesis [18] and *Acsl3*, which has been shown to transcriptionally regulate lipogenic genes [14], were coordinately up-regulated by FR in iWAT, gWAT, and liver when *de novo* lipogenesis is increased (**Fig. 5**). *Acss2* and *Acsl3* were also down-regulated in the liver by KD and Fasting, when *de novo* lipogenesis is reduced. These data suggest that these two acyl-CoA synthetases are co-regulated to coordinate fluxes in *de novo* lipogenesis. This coordinated regulation is potentially mediated by *Acsl3* regulation of SREBP for which *Acss2* is a target [14,18].

The long-chain acyl-CoA synthetase 1 (Acsl1) was thought to activate fatty acids for triglyceride synthesis primarily due to its up-regulation during 3T3-L1 adipocyte differentiation and because its over-expression results in triglyceride accumulation in several models [19–21]. However, the generation of an Acsl1 deficient mouse provided evidence that this enzyme channels fatty acids towards fatty acid oxidation in adipose and heart, with little effect on fatty acid anabolic flux [22,23]. In agreement, *Acsl1* mRNA was up-regulated in fasting liver and kidney, however its mRNA was unchanged across conditions in white adipose tissue, a tissue for which the role and requirement for fatty acid oxidation remain unclear. To date, it remains unknown which of the ACSL/ACSVL family members plays a pivotal role in triglyceride (TAG) formation. From this screen we can speculate that *Acsl4*, *Acsl5*, or *Fatp6* would be involved in TAG synthesis because these enzymes are up-regulated in HFD fed gWAT (**Fig. 5**). *Acsvl2/Fatp6* was described as a heart-specific enzyme in humans, however, these data showed that *Acsvl2/Fatp6* mRNA is highly expressed in mouse adipose tissue (**Fig. 3**).

*Them1/Acot11* was most highly expressed in brown adipose tissue and the loss of this enzyme in mice results in resistance to high-fat diet induced obesity, fatty liver, and insulin resistance [24]. *Acot11* was up-regulated in adipose and liver during conditions that increase lipid anabolic processes, specifically *Acot11* was up-regulated 10- and 34-fold in FR gWAT and iWAT, respectively, and 5-fold and 13-fold in liver during FR and HFD, respectively (**Fig. 4**). These data suggest that Acot11 may play a role in lipid anabolism, perhaps by antagonizing lipid catabolism. This agrees with the increased rates of fatty acid oxidation in the Acot11 knockout mouse [24].

In summary, our expression analysis has revealed the tissue-specific enrichment of many of the ACS and ACOT enzymes likely reflecting tissue-specific metabolic control. We demonstrate that the genetically clustered type I ACOTs are largely regulated in concert, whereas the genetically clustered ACSM subfamily showed distinct regulatory patterns. These data confirmed previous findings, revealed many new regulatory modes, and suggest concerted regulation of these enzyme families in order to coordinate fatty acid metabolic flux.

### Tissue-specific transcriptional-translational coordination of cytosolic long-chain acyl-CoA thioesterases

**Fig 6:**
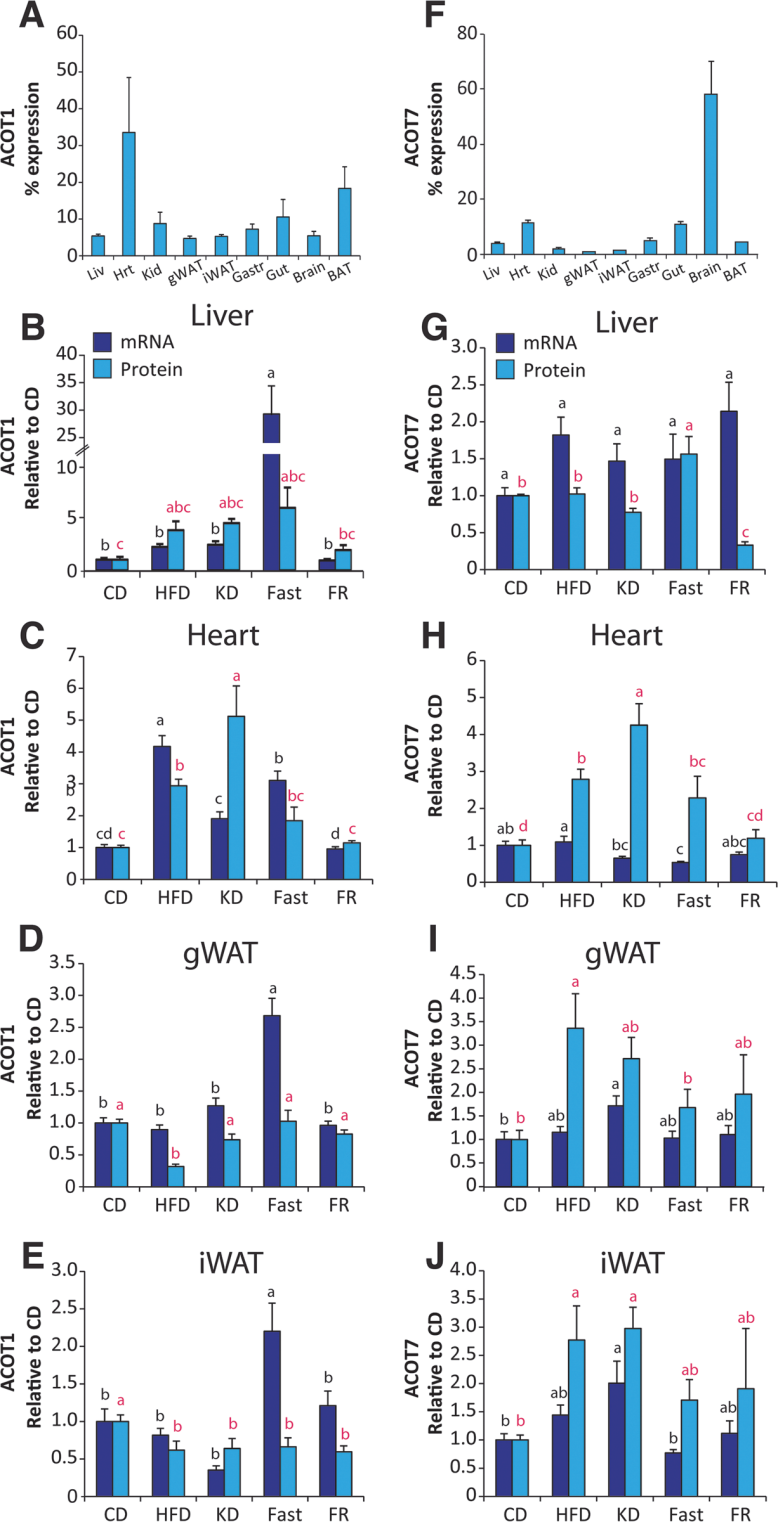
Tissue-specific posttranscriptional regulation of Acot1 and Acot7. Protein abundance for (**A**) Acot1 and (**F**) Acot7 across tissues expressed as percent of total protein visualized, n = 3. Gene mRNA and protein abundance across dietary conditions, relative to control diet group, in (**B,G**) liver, (**C**,**H**) heart, (**D**,**I**) gonadal white adipose tissues (gWAT), and (**E**,**J**) inguinal white adipose tissue (iWAT) for Acot1 and Acot7, respectively, n = 5–6. Significant differences were determined by Tukey multiple comparison tests (p<0.05) after one-way ANOVA. Images of blots are provided in **S3 Fig.**

To understand if transcriptional differences translate into changes in protein abundance, we performed Western blotting on a subset of samples. We focused on Acot1 and Acot7 because these enzymes are both cytosolic and long-chain acyl-CoA preferring, however, they are structurally distinct [4], have unique tissue distribution (**Fig. 2**) and transcriptional regulation (**Fig. 4**). Across tissues, we observed high abundance of *Acot1* mRNA in liver, kidney, heart, and muscle (**Fig. 2**), and the expression was confirmed at the protein level by western blot analysis across these tissues, as well as in brown adipose (**Fig. 6A and S3 Fig.**). The type I ACOTs, including Acot1, are PPARα targets that we found to be up-regulated most prominently by fasting across multiple tissues [16,25,26]. The up-regulation of ACOT enzymes by PPARα, a transcription factor that promotes fatty acid oxidative metabolism, is seemingly paradoxical due to the fatty acid metabolic antagonism performed by the thioesterase reaction. Thus, to confirm that the transcriptional regulation did indeed increase Acot1 at the protein level, we analyzed its expression by Western blot. We found that in liver and heart tissue, the up-regulation of *Acot1* mRNA correlated with protein abundance as seen by the up-regulation of Acot1 by HFD, KD, and Fasting (**Fig. 6B,C and S3 Fig.**). These data confirm a seemingly enigmatic up-regulation of a cytoplasmic thioesterase during times of increased FA metabolic flux. Conversely, we observed an overall transcriptional down-regulation of type I ACOTs during times of increased FA metabolic anabolism, such as during HFD and KD in white adipose tissue. To confirm this regulation we determined the protein content of Acot1 in white adipose tissue across dietary conditions. We find that Acot1 protein is down-regulated during nutritional excess, HFD and KD as suggested by the mRNA data (**Fig. 6D,E and S3 Fig.**). However, during fasting when PPARα activity is increased we observed an increase in *Acot1* mRNA but no increase in Acot1 protein abundance (**Fig. 6D,E**). These data show tissue-specific regulation of Acot1 at both the transcriptional and post-transcriptional level.

Acot7 is highly enriched in neurons [27]. Here we found that *Acot7* mRNA was highly abundant in brain, soleus muscle, and heart (**Fig. 2**). Acot7 protein, however, was highly abundant in the brain with comparatively lower expression observed in all other tissues (**Fig. 6F and S3 Fig.**). Despite low expression of Acot7 in tissues outside of the CNS, its mRNA was regulated by our dietary conditions across several tissues (**Fig. 4**). To determine if Acot7 protein was indeed regulated in tissues outside of the CNS, we determined its protein abundance across conditions in liver, heart, and white adipose. In liver and heart *Acot7* mRNA and protein abundance did not correlate (**Fig. 6G,H**). In heart Acot7 protein, but not mRNA, was increased by KD and HFD, consistent with previous reports [28]. In liver *Acot7* mRNA is increased by HFD and FR, yet Acot7 protein is increased by fasting and decreased by KD and FR. Conversely, *Acot7* mRNA and protein correlated in adipose tissue and were up-regulated by HFD and KD (**Fig. 6I,J**). Together, these data show that the ACOT genes are regulated by transcriptional and post-transcriptional mechanisms in a tissue-dependent manner.

### Increasing cytoplasmic acyl-CoA thioesterase activity affects fatty acid metabolism in a tissue-specific manner

**Fig 7:**
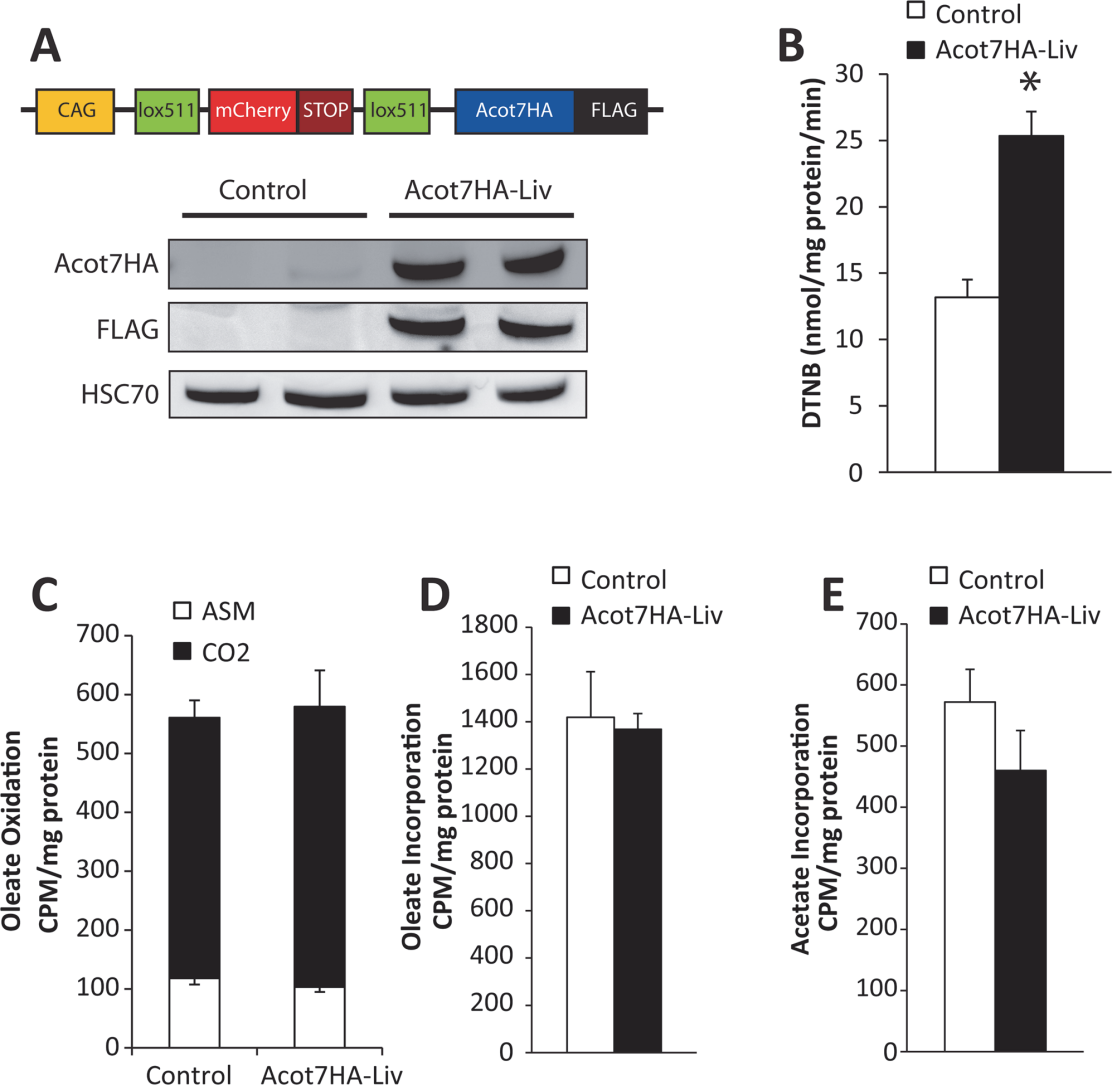
Development of a transgenic mouse model with a conditional tissue-specific and cytoplasmically targeted long-chain acyl-CoA thioesterase. (**A**) Transgenic construct schematic and representative western blot confirming Acot7HA-FLAG overexpression in liver. (**B**) Thioesterase activity for oleoyl-CoA in liver lysate from control and Acot7HA-Liv transgenic mice, n = 5–7. Overnight fasted control and Acot7HA-Liv liver slice rates of (**C**) 14C-oleate oxidation, (**D**) 14C-oleate incorporation into complex lipids, and (**E**) 3H-acetate incorporation into lipids, n = 5–7. Data represent mean ± SEM, * represent p≤0.05 by Student’s t-test.

To further understand the functional consequence and tissue-specific role of cytosolic thioesterase-induced fatty acid deactivation, we designed a transgenic mouse model to conditionally over-express an active and cytoplasmic long-chain acyl-CoA thioesterase. We chose Acot7 as a model thioesterase because its enzymatic and regulatory properties have been previously characterized including a crystal structure [4,5,29–31]. Acot7 is a typical hotdog fold type II thioesterase that encodes two thioesterase domains per polypeptide, one of which is inactive yet binds substrate resulting in a “half-of-sites inhibition” regulation of the enzyme [29]. Engineering the inactive site to an active thioesterase doubles the enzyme activity [29]. We capitalized on this discovery by mutating the mouse Acot7 E39D and T198N residues thereby generating a hyperactive Acot7 expressing two active thioesterase domains. This hyperactive Acot7 (Acot7HA) was cloned downstream of a floxed red fluorescent protein, mCherry, in a manner that allowed Cre recombinase-dependent excision of the mCherry to cause a shift in the translational reading frame allowing expression of Acot7HA (**Fig. 7A**). The Acot7HA vector was used to generate transgenic mice which were subsequently crossed with albumin-driven Cre expressing mice to yield liver-specific Acot7HA expressing mice (Acot7HA-Liv). We confirmed Cre-dependent Acot7-HA expression in liver resulting in robust protein expression and ~2-fold greater cytosolic long-chain acyl-CoA thioesterase activity (**Fig. 7A,B**). To determine how doubling cytosolic long-chain acyl-CoA thioesterase activity altered fatty acid metabolic flux, we traced radiolabeled substrates in liver slices from control and Acot7HA-Liv mice. Surprisingly, increased cytosolic thioesterase activity under these conditions did not reduce the rate of fatty acid metabolic flux into either catabolic or anabolic products (**Fig. 7C-E**).

**Fig 8:**
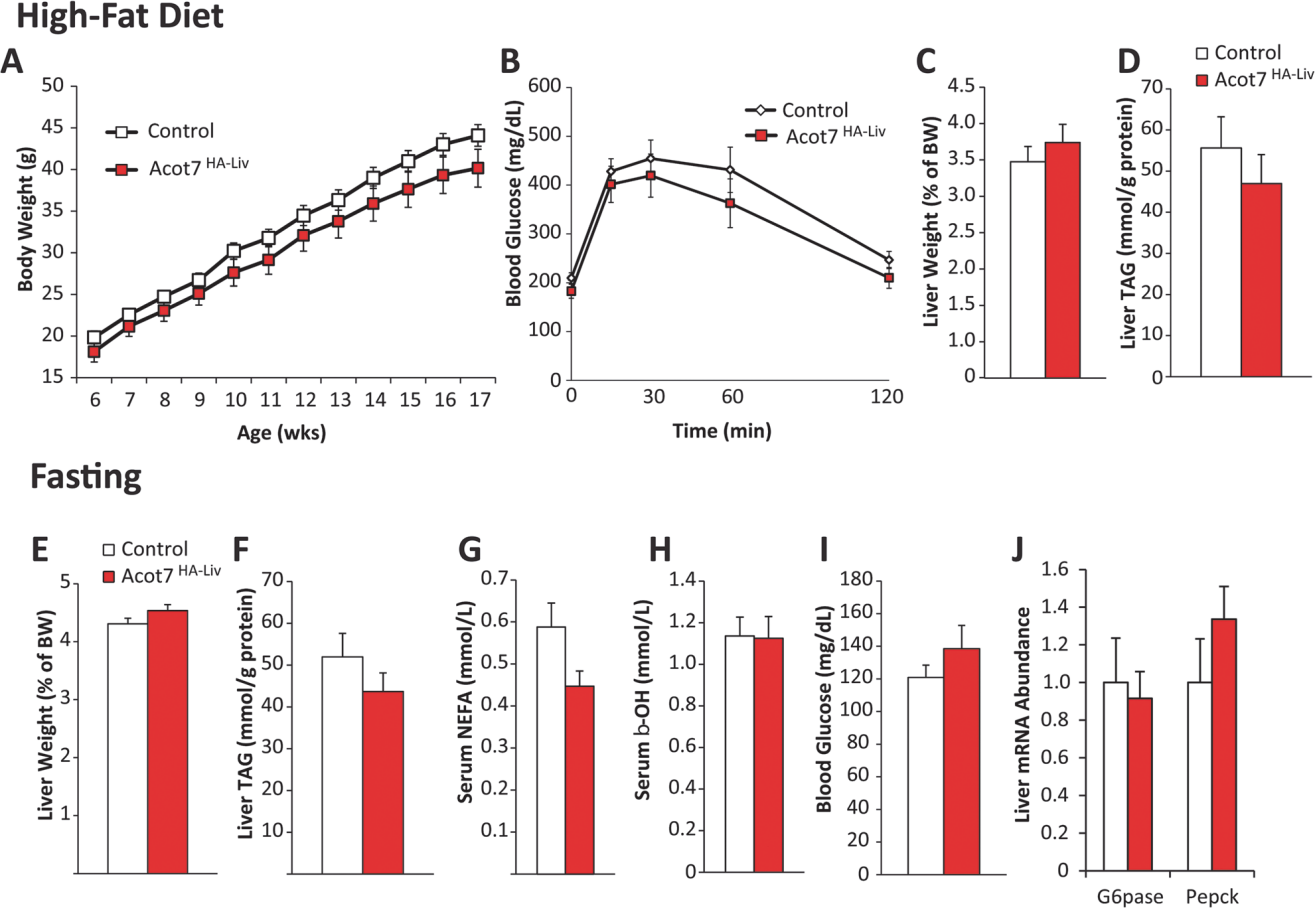
Doubling of hepatic cytoplasmic long-chain acyl-CoA thioesterase activity does not alter liver fatty acid metabolism. Control and Acot7HA-Liv (**A**) weight gain, (**B**) response to glucose tolerance test, (**C**) liver weight, and (**D**) liver triacylglycerol (TAG) in response to high-fat diet feeding for 11 weeks, n = 8–12. Control and Acot7HA-Liv (**E**) liver weight, (**F**) liver TAG, (**G**) serum non-esterified fatty acids (NEFA), (**H**) serum β-hydroxybutyrate, (**I**) blood glucose, and (**J**) liver mRNA abundance of gluconeogenic genes in response to overnight fasting (18 hours), n = 7–11. Data represent mean ± SEM.

To determine if increased cytosolic thioesterase activity could effectively antagonize fatty acid metabolism *in vivo*, we challenged Acot7HA-Liv mice with conditions that increase liver fatty acid metabolic flux. To induce fatty liver and insulin resistance, the Acot7HA-Liv mice were challenged with a high-fat (60%) diet for 12 weeks. The Acot7HA-Liv mice gained weight, became insulin resistant, and developed fatty liver similar to controls (**Fig. 8A-D**). Diet-induced fatty liver is a chronic process. To examine an acute hepatic fatty acid challenge, we subjected Acot7HA-Liv mice to an overnight fast. During fasting, the liver utilizes fatty acid oxidation to produce ketones and to drive gluconeogenesis. Under this acute metabolic challenge the Acot7HA-Liv mice had similar triglyceride content, mRNA abundance of gluconeogenic genes, and circulating ketones and glucose relative to their littermate controls (**Fig. 8E-J**). Therefore, increased liver cytosolic long-chain thioesterase activity is not sufficient to prevent diet-induced fatty liver.

**Fig 9:**
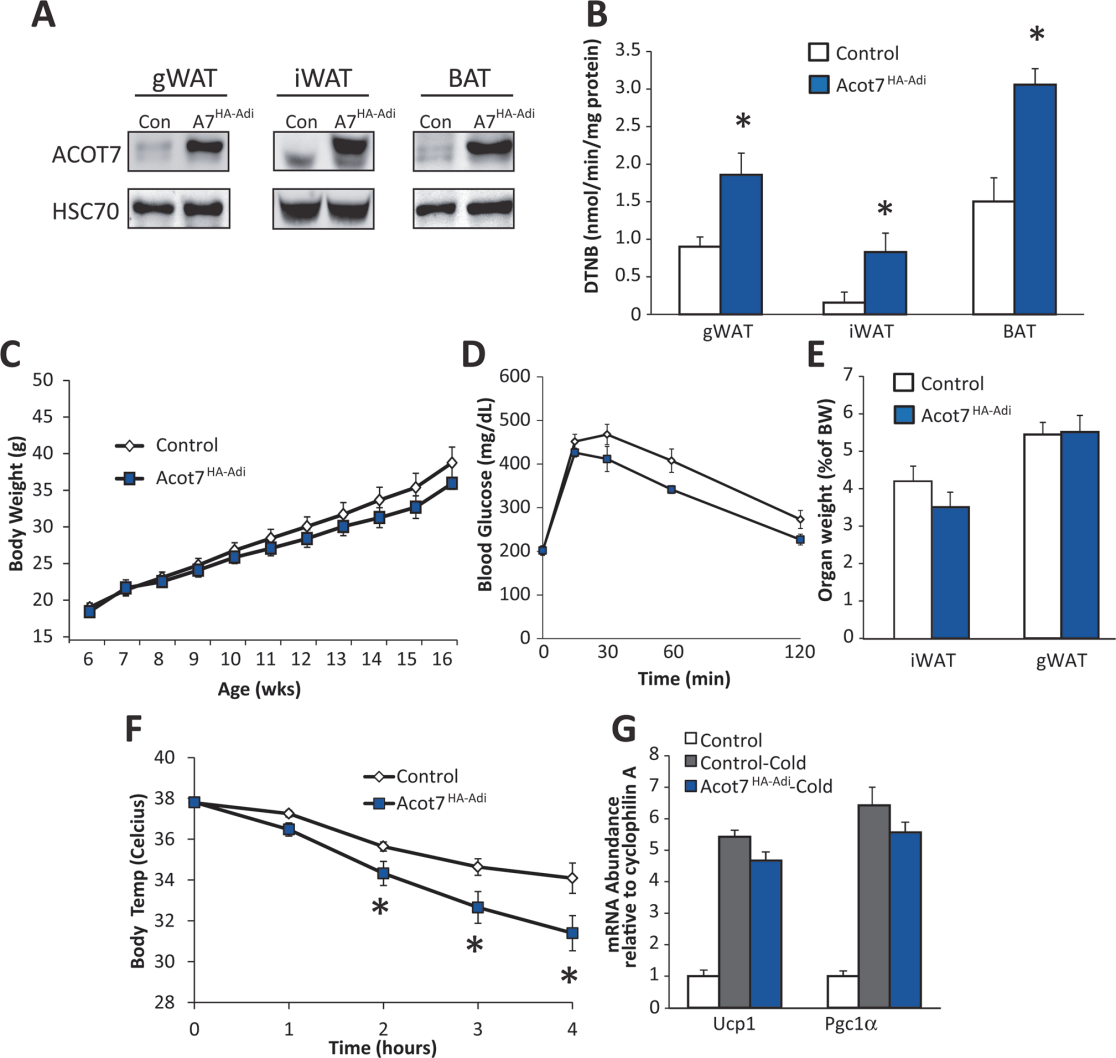
Increased adipocyte cytoplasmic long-chain acyl-CoA thioesterase activity inhibits cold-induced thermogenesis but does not protect against diet-induced obesity. **A**) Representative Acot7 western blot and (**B**) thioesterase activity for oleoyl-CoA in control and Acot7HA-Adi gonadal adipose (gWAT), inguinal adipose (iWAT), and brown adipose (BAT), n = 3–12. Control and Acot7HA-Adi (**C**) weight gain, (**D**) response to glucose tolerance test, and (**E**) inguinal and gonadal adipose weight in response to high-fat diet feeding for 11 weeks, n = 3–12. Control and Acot7HA-Adi (**F**) body temperature and (**G**) mRNA abundance of adrenergic genes in brown adipose in response to acute 4 hour cold (4°C) exposure, n = 8–10. Data represent mean ± SEM, * represent p≤0.05 by Student’s t-test.

Adipose tissue expansion during obesity is a potential target to combat obesity and diabetes, therefore to determine if cytosolic thioesterase activity could reduce fatty acid accretion into adipocyte triglyceride to thwart the onset of diet-induced obesity and insulin resistance we expressed Acot7HA in adipose tissue specifically using the Adiponectin-Cre mouse (Acot7HA-Adi). The Acot7HA-Adi mice had ~2-fold greater long-chain thioesterase activity in visceral (gWAT), subcutaneous (iWAT), and brown adipose tissue (BAT) (**Fig. 9A,B**). The Acot7HA-Adi and littermate control mice were fed a high-fat (60%) diet for 12 weeks. The Acot7HA-Adi mice gained weight, responded to a glucose tolerance test, and accumulated adipose mass similar to their control littermates (**Fig. 9C-E**). Therefore, doubling adipose thioesterase activity was not sufficient to inhibit diet-induced obesity or insulin resistance.

To determine if increased cytosolic long-chain acyl-CoA thioesterase activity antagonized the rapid fatty acid metabolic flux that occurs in brown adipocytes during cold induced thermogenesis, we subjected control and Acot7HA-Adi mice to an acute 4 hour cold exposure during which the maintenance of body temperature requires fatty acid oxidation in brown adipose [32]. Here, we showed that Acot7HA-Adi mice, relative to control, had reduced body temperature during the cold challenge compared to their littermate controls (**Fig. 9F**). Adrenergic signaling remained intact in the Acot7HA-Adi mice as indicated by increased brown adipose mRNA abundance of *Pgc1*α and *Ucp1* (**Fig. 9G**). These data suggest that increased cytosolic long-chain acyl-CoA thioesterase activity in brown adipose tissue antagonizes fatty acid oxidation during an acute cold challenge rendering mice cold intolerant.

## Discussion

Aberrant lipid metabolism is a cornerstone of many diseases, such as obesity, diabetes, and cardiovascular disease [33]. The initial and required step for lipid catabolic and anabolic metabolism is the formation of an acyl-CoA that serves as an acyl donor for diverse acyltransferases. The dysregulation of this step can lead to deleterious physiological outcomes. The overexpression of acyl-CoA synthetases in cell culture and animal models results in increased acyl-CoA formation, a toxic product that is ultimately cleared by either thioesterase action or is channeled into lipid synthesis [2,34]. The cardiac-specific overexpression of Acsl1 ultimately results in cell death, physiological dysfunction, and reduced lifespan [35]. The loss of Them5, a long-chain preferring mitochondrial thioesterase, in mice causes fatty liver by disrupting cardiolipin remodeling and mitochondrial function [36]. These reports demonstrate the importance of a tightly controlled acyl-CoA concentration. To that effect, there are 26 human acyl-CoA synthetase enzymes and 12 acyl-CoA thioesterases responsible for the formation and breakdown of acyl-CoA. The sheer number of these enzymes exemplifies the critical and complex mechanisms that control the initiation of fatty acid metabolism. Also, other hydrolases that do not fit into these families may additionally regulate fatty acid flux [37–39]. Here, we have investigated the concerted transcriptional regulation of the canonical acyl-CoA synthetase and thioesterase enzymes across metabolic tissue under various nutritional stresses. We show diverse patterns of expression and transcriptional control for these enzymes that likely act in a coordinated fashion to control fatty acid metabolic flux.

While databases such as bioGPS and RefExA provide gene expression data across tissues, not all tissues are profiled. For example, unlike the widely used databases, we separated the red soleus from the white plantaris muscle to discover rather striking differential expression between the two muscles. For example, *Acot1*, *Acot2*, *Acot6*, *Acot7*, *Acsf4*, *Acsl1*, *Acsl4*, *Fatp1*, and *Fatp4* are more abundant in the red soleus muscle than in the white plantaris muscle (see **Fig. 2 and Fig. 3**). Furthermore, the metabolic perturbations regulated genes differentially between the red and white muscle, likely reflecting differences in metabolic activity. Additionally, the popular databases do not distinguish between the different white adipose depots. Here we analyze gene expression in visceral (gonadal) and subcutaneous (inguinal) adipose tissue to show differential expression of these genes between the two depots (see **Fig. 2 and Fig. 3**), as well as distinct transcriptional control by metabolic perturbations in an adipose-depot dependent manner (see **Fig. 4 and Fig. 5**).

Several ACS and ACOT enzymes have been investigated to determine their individual physiological role. Because there are a large number of these enzymes with converging substrate preferences, it is highly likely that these enzymes do not act individually to control lipid metabolism but rather act coordinately. Indeed, the loss of Acsl1, showed little effect on fatty acid metabolism in the liver [40]. However, this effect was shown to be tissue-dependent with the loss of Acsl1 severely inhibiting fatty acid oxidation in muscle and adipose tissues [22,23]. These data suggest tissue-specific coordinated regulation of fatty acid activation.

The role of thioesterases in regulating fatty acid metabolism remains unclear. The reaction reverses the energetically demanding formation of acyl-CoAs in an apparent futile cycle. Logically, it would seem that thioesterases regulate excessive metabolism of fatty acids in a cell-and organelle-specific manner. However, it remains unclear if and how these thioesterases regulate the metabolic fate of a fatty acid. Several thioesterase knockout mouse models have been recently reported. The loss of Acot7 [41], Acot11/Them1 [24], Acot13/Them2 [42], and Acot15/Them5 [36] result in phenotypes ranging from increased neurodegeneration to altered susceptibility to high-fat diet induced obesity, fatty liver, and insulin resistance through unknown mechanisms that unexpectedly altered whole body metabolism. Overexpression of a cytosolic thioesterase, Acot1 in the heart reduced cardiac oxidative stress and dysfunction potentially by altering transcriptional control [43]. Overexpression of a mitochondrial thioesterase, Acot2 in the liver increased fatty acid oxidation and ketogenesis through mechanisms that remain unclear [44]. Further investigation of these mouse models should ultimately lead to a more detailed understanding of how these enzymes regulate lipid metabolism. Here we have shown that overexpression of a hyperactive cytosolic long-chain acyl-CoA thioesterase does not alter fatty acid flux in the liver potentially due to the enzyme’s inability to coordinate metabolic flux with endogenous hepatic enzymes; however, in adipose tissue Acot7 overexpression impaired cold-induced thermogenic capacity, supporting an antagonistic role for cytosolic thioesterase activity in brown adipose tissue.

Surprisingly, we found that seven of the Acots were significantly up-regulated in cold-exposed brown adipose tissue, suggesting an important regulatory role for thioesterase activity during times of increased fatty acid catabolism. However, it is unclear whether hydrolyzing acyl-CoAs under these conditions serves simply to antagonize fatty acid oxidation by reducing substrate availability, as seems to be the case in animals expressing the Acot7HA transgene in adipose tissue. Conversely, thioesterase activity may actually promote enhanced oxidation by protecting against acyl-CoA oversupply, recycling coenzyme A, or altering the transcriptional regulation of fatty acid metabolic genes, as is suggested by Acot2 overexpression in the liver [44].

Subcellular compartmentalization likely impacts the functional consequences of thioesterase activity. It has been recently shown that adenoviral overexpression of the mitochondrial Acot2 in mouse liver is sufficient to increase whole body fatty acid oxidation, suggesting that Acot2 may prevent the accumulation of fatty acid oxidation intermediates in the mitochondria [44]. At the nucleocytoplasmic level, changes in cytosolic ACOT activity may impact the availability of acyl-CoAs for transcriptional regulation. Acyl-CoAs have been shown to regulate gene expression in *E*.*coli* and in *S*.*cerevisiae* [45,46], and there is evidence that acyl-CoAs can bind mammalian transcription factors such as nuclear thyroid hormone receptor and hepatic nuclear factor-4α [47,48]. Additionally, a variety of long-chain acyl-CoAs and unsaturated fatty acids have been identified as high-affinity endogenous PPARα ligands [49]; although, the physiological implications of transcriptional modulation by acyl-CoAs or free fatty acids are not fully understood. Many thioesterases are themselves regulated by these same transcription factors suggesting transcriptional feedback or feed-forward regulatory mechanisms.

Here we show how ACS and ACOT mRNA abundance is regulated in a tissue-dependent manner in defined physiological contexts, providing insight into transcription factor dominance over these genes. For example, we show that under conditions of increased PPARα activity, specifically during fasting, the transcript of the PPARα target *Acot1* [25] is significantly increased in all tissues with the exception of the brain. Interestingly, however, *Acsl1* was shown to be regulated by PPARα [25] yet its transcript is only up-regulated by fasting in the liver, kidney and gut, suggesting complex and differential regulation of PPAR target genes within these gene families. These data suggest that the specific transcription factors modulating thioesterase and synthetase gene expression are not the sole determinants of regulating transcript level in a physiological context. In this case we have shown that for Acot1 and Acot7 the transcript abundance may not reflect protein abundance in all tissues, suggesting tissue-specific post-transcriptional regulation in addition to complex and coordinated transcriptional control.

Both ACOT and ACS enzymes are reported to form multimers [29,50–53]. These reports suggest both homo- and heteromerization. For example, Fatp1 is reported to form a homodimer, a heterodimer with Acsl1, and a multimer of Fatp1 with Acsl1 [19]. Multimerization of all members of the ACS and ACOT gene families has not been thoroughly investigated, nor is it understood how the multimerization alters the function of the enzymes. Thus, the regulation of one synthetase or thioesterase may alter the activity of another enzyme with which it forms a multimer in a manner that remains unexplored. Here we present the transcriptional regulation of all of these enzymes to serve as a foundational reference from which extrapolation of the functional consequences of these enzymes as independent proteins, as dimers, as antagonists of one another, and as regulators of similar pathways can be determined.

The evolutionary diversification of the ACS and ACOT enzyme families suggests the importance of tissue-specific regulation of acyl-CoA metabolism. Divergence of closely related acyl-CoA-metabolizing enzymes may allow for specialized function by altering the subcellular location or substrate preferences. For example, an evolutionary divergence allowing for distinct function in lipid metabolism has been described for the type I ACOTs (Acot1-6), of which Acot1 is cytoplasmic, Acot2 is mitochondrial, and Acot3-Acot6 are peroxisomal with distinct substrate preferences [4,54]. The divergent role of these enzyme families is controlled in part by transcriptional and post-transcriptional tissue-specific regulatory elements. In this way, the evolutionary history of these acyl-CoA-metabolizing enzymes supports the notion of both distinct and coordinated regulation of the tissue-specific proteome.

The acyl-CoA synthetases and acyl-CoA thioesterases are poised at an important regulatory node in fatty acid metabolism. A better understanding of how these enzymes are regulated under different dietary conditions and in a tissue-specific manner will provide insight into the basic regulatory mechanisms of whole-body fatty acid metabolism. Additionally, the diversity of expression patterns of these enzymes across tissues allows for pharmacological interventions targeting a particular ACS or ACOT to provide therapeutic options against various metabolic syndromes.

## Materials and Methods

### Animals

C57Bl/6J male mice purchased from Jackson Laboratory were used for nutritional modulation experiments. These male mice were randomly placed on the specified diets for 12 weeks from age 8 to 20 weeks, n = 10–15. The CD (AIN-76A) and HFD (F6690, 60% in %kcal) are stock diets from BioServ, (Flemington, NJ) the KD (F6689 Fat: Protein+Carbohydrate, 4:1) was custom made to match fat and protein source with HFD (see **S1 Table**). At the end of the dietary treatment, mice were fasted for 2–4 hours for the CD, HFD, and KD fed groups, fasted overnight (16–18 hours) for the fasted group, or fasted for 14 hours and refed for 12 hours for the FR group before tissue harvest. Mice were anesthetized with isoflurane and euthanized by decapitation. All tissues were harvested between 10AM and 1PM. The Acot7HA transgenic mice were generated by pronuclear injection on the C57Bl/6J background. The Cre inducible transgenic vector was generated as we have previously described [55,56]. Acot7HA transgenic mice were bred to either Albumin-Cre (Jax Labs, Bar Harbor, ME) [57] or Adiponectin-Cre (Jax Labs) [58] C57Bl/6 mice and placed on a high-fat (60%) diet D12692 (Research Diets, New Brunswick, NJ) from ages 6 wks to 18 wks. Control littermates were a combination of mice expressing either the Cre or Acot7HA or neither. Intraperitoneal glucose tolerance tests were performed after 10 weeks on the diet at a dose of 2 g/kg and blood glucose was determined using a glucometer at 15, 30, 60, and 120 minutes after the glucose injection. Insulin tolerance tests were performed after 11 weeks on the diet at a dose of 0.75U/kg and blood glucose was determined using a glucometer at 15, 30, 60, and 90 minutes after the insulin injection. For all thermogenesis experiments, singly-housed mice were placed in a 4°C environment without food. Temperature was measured hourly with a rectal probe thermometer (BAT-12, Physitemp, Clifton, NJ).

### Ethical Statement

All procedures were performed in accordance with the National Institutes of Health Guide for the Care and Use of Laboratory Animals and under the approval of the Johns Hopkins Medical School Animal Care and Use Committee.

### Serum metabolites

Blood glucose was determined by glucometer (NovaMax, Billerica, MA). Blood was collected in 5–10% 0.5M EDTA and plasma NEFA (Wako, Richmond, VA), glycerol/triacylglycerol (Sigma, St. Louis, MO), and beta-hydroxybutyrate (StanBio, Boerne, TX) was determined by colorimetric assay per manufacturer’s instruction. Liver total lipids were extracted with chloroform/methanol via Folch method [59], the chloroform phase was dried down by speed vac and resuspended in tert-butanol:methanol:Triton X-100 (3:1:1 ratio by volume) and lipids were quantified using colorimetric glycerol/triacylglycerol assay (Sigma). Total acyl-CoA thioesterase was measured with 50 μM oleoyl-CoA (Sigma) and 10 mM 5,5’-dithiobis(2-nitrobenzoate) (DTNB, Sigma) with CoA standards run in parallel in either total homogenate or cytosol fractions (25–50 μg protein).

### RNA isolation and analysis

RNA was isolated using standard Trizol method followed by RNeasy Mini Plus Kit (Qiagen, Valencia, CA) according to manufacturer’s instructions. cDNA was synthesized using Applied Biosystems (Carlsbad, CA) High Capacity cDNA RT Kit. RNA was quantified using Nano Drop. RT-PCR was performed using BioRad (Hercules, CA) SsoAdvanced SYBR Green master mix with primers specific to the genes of interest (see primer sequences in **S3 Table**). Outliers were removed using ROUT method with GraphPad Prism 6 software. All data was normalized to the average CT value for *Rpl22*, *βeta-actin*, *Gapdh*, and *18S*. Data is expressed as 2^dCT and relative to the control diet fed group.

### Protein Analysis

Tissue total lysates were prepared in lysis buffer (50mM Tris-HCl, 150mM NaCl, 1mM EDTA, 1% Triton X-100) and 20–30 μg of protein were separated by SDS PAGE electrophoresis and transferred to nitrocellulose membranes. Acot1 (ab133948, Abcam, Cambridge, MA), Acot7 (affinity purified antibody [41]), and Hsc70 (sc-59570, Santa Cruz, Dallas, TX) coupled with anti-rabbit Cy5 (Invitrogen, Grand Island, NY) or anti-mouse Cy3 (Invitrogen) were visualized with Alpha Innotech MultiImage III and quantified using Alpha Innotech FluorChem Q Software (Santa Clara, CA), and data was normalized to Hsc70 expression.

### Liver slice studies

Liver slices were collected from overnight fasted mice and the left lobe of the livers were immediately sliced into 350μm sections using the McIlwain Tissue Chopper (Redding, CA). Slices were incubated in HEPES buffer with 0.5mM L-Carnitine, 0.2% BSA, 25μM Glutamine, with either 1–14Coleate (Perkin Elmer, Akron, OH), U-14Cglucose, or 3Hacetate in a 37°C water bath for 1–3 hours with gentle shaking. Rates of CO_2_ and acid soluble metabolites (ASM) production from 1–14Coleate were determined using incubation chambers containing a center well filled with filter paper and sealed with a rubber stopper. Carbon dioxide was trapped by adding 200 μl 70% perchloric acid to the reaction mixture and 300 μl of 1 M NaOH to the center well, and incubating the samples at 55°C for 1 hr. The filter paper was then placed into scintillation fluid and counted. The acidified reaction mixture was incubated overnight at 4°C and centrifuged at 4,000 rpm for 30 min before aliquots of the supernatant were counted for 14C-labeled ASM. All slice experiments were performed with 3–4 slices per assay per mouse and averaged over 3 independent experiments. Fatty acid incorporation was measured by incubating slices with 1–14Coleate for 16 hrs, after which slices were washed 3 times with 1% BSA-PBS. Total lipid was extracted from slices that were manually homogenized in glass mortal-pestle and extracted into CHCl3 [60], mixed with scintillation fluid and counted. For fatty acid synthesis, liver slices were labeled with 0.5 μCi of 3H-acetate (Perkin Elmer) for 1 hr. Total lipids were extracted with chloroform/methanol via Folch method [59] and radioactivity was counted via liquid scintillation.

### Statistics

Data was compared by Student’s t-test or by one-way ANOVA and means were compared using Tukey’s HSD post-hoc analysis with JMP 11 statistical software.

## Supporting Information

S1 Fig One-way ANOVA of nutritional modulation of ACOT enzymes. One-way ANOVA data table comparing all pairs by Tukey’s post-hoc analysis. Similar letters indicate non-significance for control diet (CD), high-fat diet (HFD), ketogenic diet (KD), overnight fasted (Fast), or overnight fasted followed by 12-hour refeeding (FR), mice (n = 6–8).
(TIF)

S2 Fig One-way ANOVA of nutritional modulation of ACS enzymes. One-way ANOVA data table comparing all pairs by Tukey’s post-hoc analysis. Similar letters indicate non-significance for control diet (CD), high-fat diet (HFD), ketogenic diet (KD), overnight fasted (Fast), or overnight fasted followed by 12-hour refeeding (FR), mice (n = 6–8).
(TIF)

S3 Fig Tissue-specific posttranscriptional regulation of Acot1 and Acot7. **A**) Representative western blot images for Acot7, Acot1, and Hsc70 across tissues. **B**) Representative western blot images for Acot1, Acot7, and Hsc70 for control diet (CD), high-fat diet (HFD), ketogenic diet (KD), overnight fasted (Fast), overnight fasted followed by 12-hour refeeding (FR) in liver, heart, gonadal white adipose tissue (gWAT), and inguinal white adipose tissue (iWAT).
(TIF)

S1 Table Diet Compositions. (DOCX)

S2 Table Gene abbreviation key. (DOCX)

S3 Table PCR primer list. (DOCX)
