# Supplementary figures and images for: Metabolic and Tissue-Specific Regulation of Acyl-CoA Metabolism
Source: PLoS One. 2015 Mar 11;10(3):e0116587. doi: 10.1371/journal.pone.0116587 (PMC4356623; doi:10.1371/journal.pone.0116587)

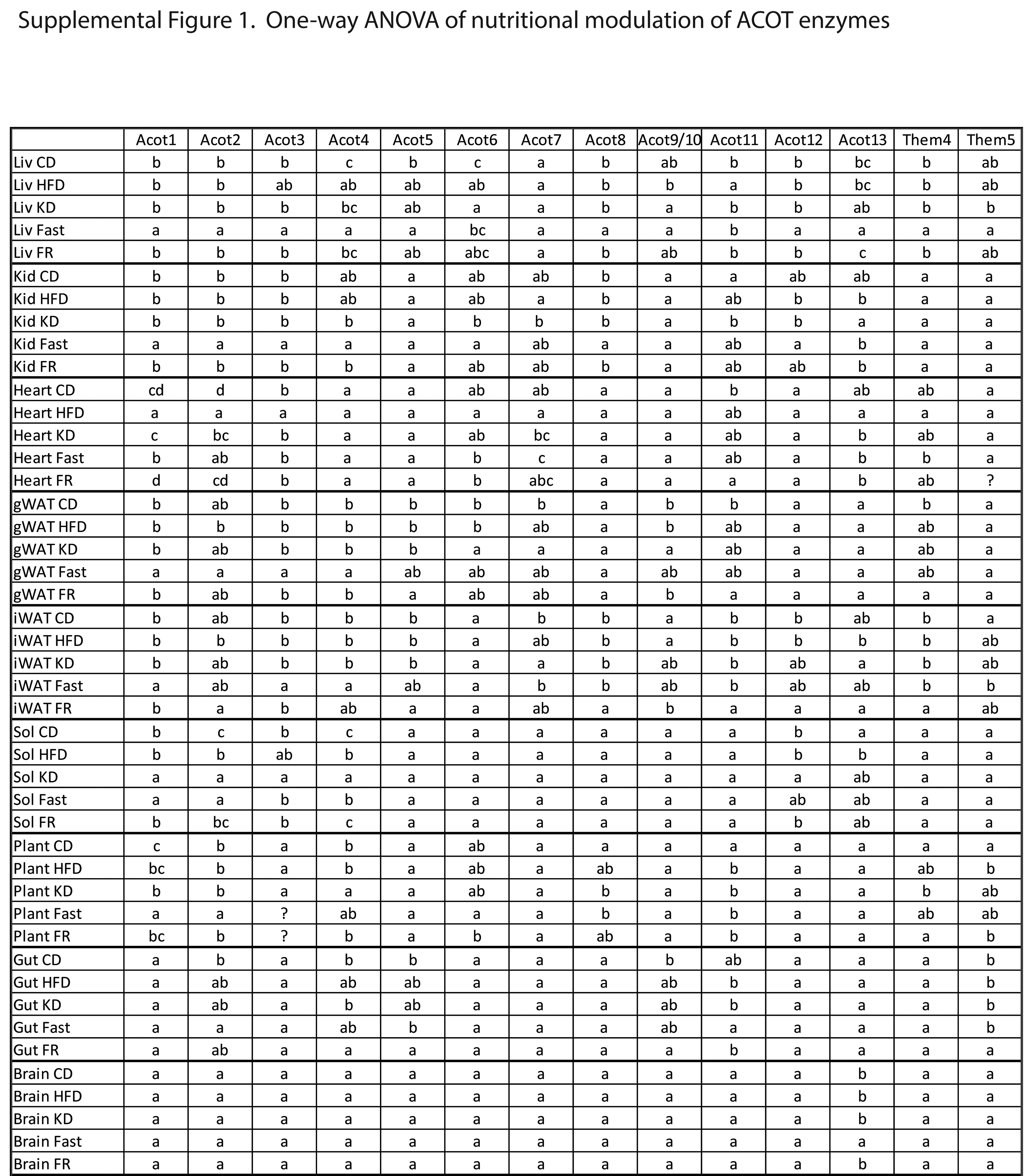

Supplement: S1 Fig — One-way ANOVA data table comparing all pairs by Tukey’s post-hoc analysis. Similar letters indicate non-significance for control diet (CD), high-fat diet (HFD), ketogenic diet (KD), overnight fasted (Fast), or overnight fasted followed by 12-hour refeeding (FR), mice (n = 6–8). (TIF) [file pone.0116587.s001.tif]

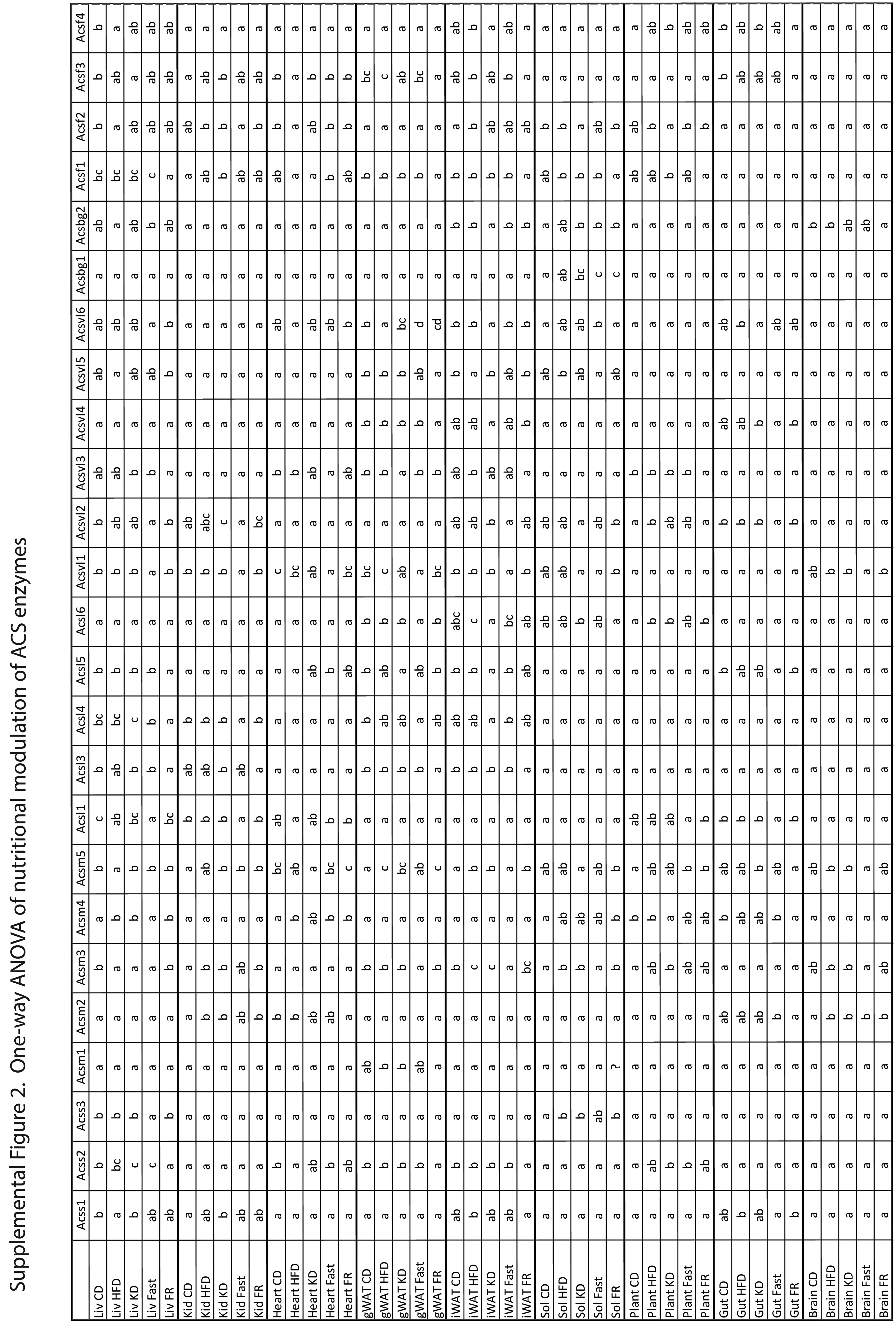

Supplement: S2 Fig — One-way ANOVA data table comparing all pairs by Tukey’s post-hoc analysis. Similar letters indicate non-significance for control diet (CD), high-fat diet (HFD), ketogenic diet (KD), overnight fasted (Fast), or overnight fasted followed by 12-hour refeeding (FR), mice (n = 6–8). (TIF) [file pone.0116587.s002.tif]

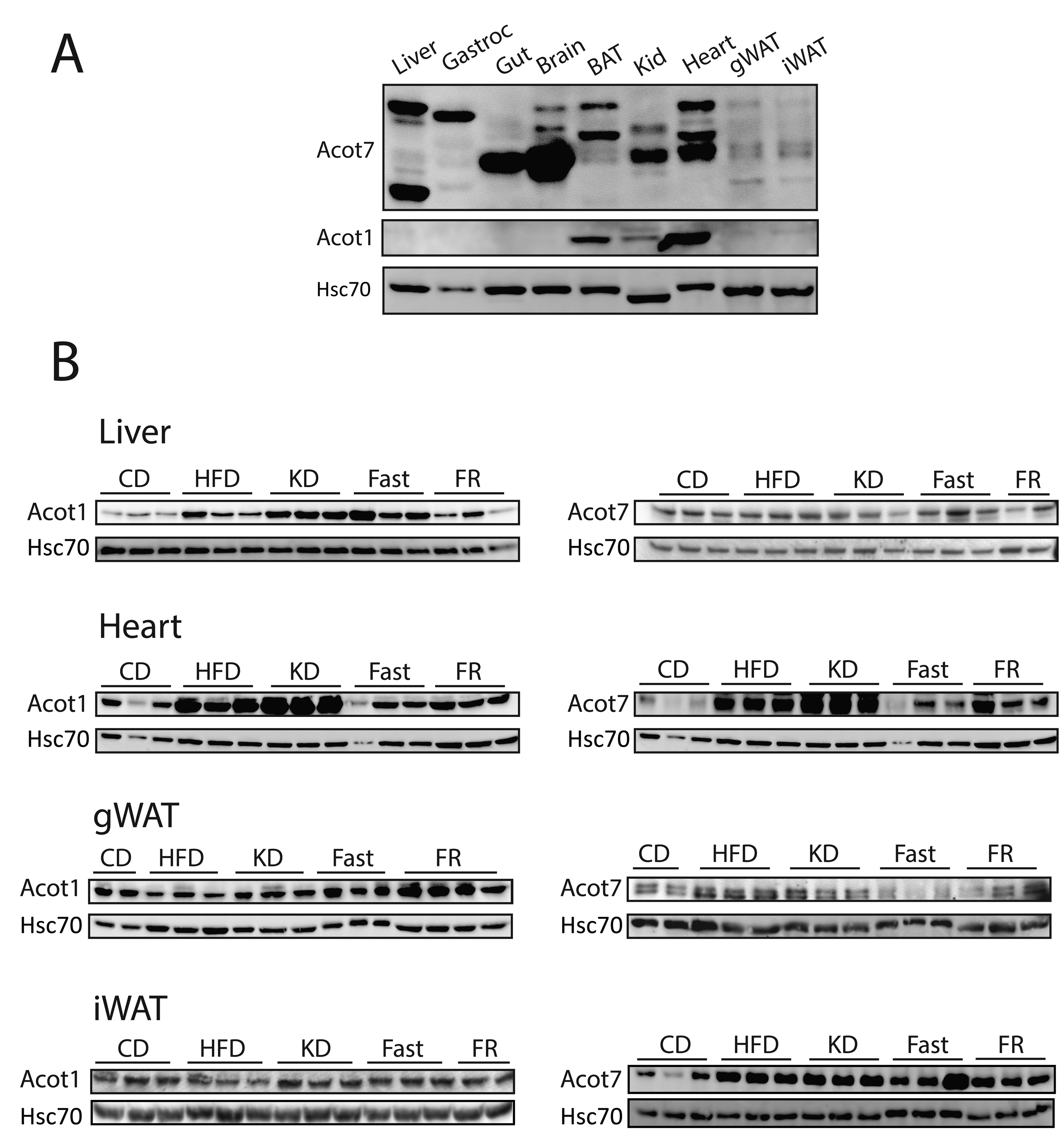

Supplement: S3 Fig — A) Representative western blot images for Acot7, Acot1, and Hsc70 across tissues. B) Representative western blot images for Acot1, Acot7, and Hsc70 for control diet (CD), high-fat diet (HFD), ketogenic diet (KD), overnight fasted (Fast), overnight fasted followed by 12-hour refeeding (FR) in liver, heart, gonadal white adipose tissue (gWAT), and inguinal white adipose tissue (iWAT). (TIF) [file pone.0116587.s003.tif]
