# Supplementary material for: Metabolic and Tissue-Specific Regulation of Acyl-CoA Metabolism
Source: PLoS One. 2015 Mar 11;10(3):e0116587. doi: 10.1371/journal.pone.0116587 (PMC4356623; doi:10.1371/journal.pone.0116587)
Supplement: S1 Table — (DOCX) [file pone.0116587.s004.docx]

**Table S1. Diet Composition**

|  | **CD**  **F1515** | **HFD**  **F6690** | **KD**  **F6689** |
| --- | --- | --- | --- |
| **Fat (% kcal)** | **5** | **61** | **90** |
| Lard (g/kg) | - | 210 | 402 |
| Corn oil (g/kg) | 50 | 50 | 96 |
| Anhydrous Milkfat (g/kg) | - | 88 | 169 |
| **Carb (% kcal)** | **65** | **21** | **1** |
| Maltodextrin (g/kg) | - | 161 | - |
| Sucrose (g/kg) | 500 | 102 | - |
| Corn starch (g/kg) | 150 | - | - |
| **Protein (% kcal)** | **18** | **18** | **9** |
| Casein (g/kg) | 200 | 258 | 165 |
| **Other** |  |  |  |
| Mineral Mix (g/kg) | 35 | 45 | 59 |
| Vitamin Mix (g/kg) | 10 | 13 | 18 |
| Choline Bitartrate (g/kg) | 2 | 3 | 3.5 |
| DL-Methionine (g/kg) | 3 | 4 | 3 |
| Cellulose (g/kg) | 50 | 65 | 86 |
