# Supplementary material for: Metabolic and Tissue-Specific Regulation of Acyl-CoA Metabolism
Source: PLoS One. 2015 Mar 11;10(3):e0116587. doi: 10.1371/journal.pone.0116587 (PMC4356623; doi:10.1371/journal.pone.0116587)
Supplement: S2 Table — (DOCX) [file pone.0116587.s005.docx]

Supplemental Table S2. Gene abbreviation key.

| **Abbreviation** | **Gene name** |
| --- | --- |
| Acss1 | Acyl-CoA synthetase short-chain family member 1 |
| Acss2 | Acyl-CoA synthetase short-chain family member 2 |
| Acss3 | Acyl-CoA synthetase short-chain family member 3 |
| Acsm1 | Acyl-CoA synthetase medium-chain family member 1 |
| Acsm2 | Acyl-CoA synthetase medium-chain family member 2 |
| Acsm3 | Acyl-CoA synthetase medium-chain family member 3 |
| Acsm4 | Acyl-CoA synthetase medium-chain family member 4 |
| Acsm5 | Acyl-CoA synthetase medium-chain family member 5 |
| Acsl1 | Acyl-CoA synthetase long-chain family member 1 |
| Acsl3 | Acyl-CoA synthetase long-chain family member 3 |
| Acsl4 | Acyl-CoA synthetase long-chain family member 4 |
| Acsl5 | Acyl-CoA synthetase long-chain family member 5 |
| Acsl6 | Acyl-CoA synthetase long-chain family member 6 |
| Fatp1 | Fatty acid transport protein 1 |
| Fatp2 | Fatty acid transport protein 2 |
| Fatp3 | Fatty acid transport protein 3 |
| Fatp4 | Fatty acid transport protein 4 |
| Fatp5 | Fatty acid transport protein 5 |
| Fatp6 | Fatty acid transport protein 6 |
| Acsbg1 | Acyl-CoA synthetase bubble gum family member 1 |
| Acsbg2 | Acyl-CoA synthetase bubble gum family member 2 |
| Acsf1 | Acyl-CoA synthetase family member 1 |
| Acsf2 | Acyl-CoA synthetase family member 2 |
| Acsf3 | Acyl-CoA synthetase family member 3 |
| Acsf4 | Acyl-CoA synthetase family member 4 |
| Acot1 | Acyl-CoA thioesterase 1 |
| Acot2 | Acyl-CoA thioesterase 2 |
| Acot3 | Acyl-CoA thioesterase 3 |
| Acot4 | Acyl-CoA thioesterase 4 |
| Acot5 | Acyl-CoA thioesterase 5 |
| Acot6 | Acyl-CoA thioesterase 6 |
| Acot7 | Acyl-CoA thioesterase 7 |
| Acot8 | Acyl-CoA thioesterase 8 |
| Acot9/10 | Acyl-CoA thioesterase 9/10 |
| Acot11 | Acyl-CoA thioesterase 11 |
| Acot12 | Acyl-CoA thioesterase 12 |
| Acot13 | Acyl-CoA thioesterase 13 |
| Them4 | Thioesterase superfamily member 4 |
| Them5 | Thioesterase superfamily member 5 |
