# Supplementary material for: Metabolic and Tissue-Specific Regulation of Acyl-CoA Metabolism
Source: PLoS One. 2015 Mar 11;10(3):e0116587. doi: 10.1371/journal.pone.0116587 (PMC4356623; doi:10.1371/journal.pone.0116587)
Supplement: S3 Table — (DOCX) [file pone.0116587.s006.docx]

**Supplemental Table 3. Real time mouse PCR primer list and corresponding Mouse Genome Informatics (MGI) identification number.**

| **Gene** | **MGI** | **Forward** | **Reverse** |
| --- | --- | --- | --- |
| **Acss1** | 1915988 | GGGACACTCCTTACCATACTGTC | GTCTCCAGAAGCTCCCTGTAAG |
| **Acss2** | 1890410 | AGGCAAACCTAAGGGTGTGG | AATGACCAGTGATCCAGCCG |
| **Acss3** | 2685720 | CAAGTTGGTTTGTGGAAGGGATGC | GACACCAGCCAGCTTAGAGAC |
| **Acsm1** | 2152200 | TCACAGTACCAGGACTGCCCTAA | AGTAGTCCAGGACATCACTTGC |
| **Acsm2** | 2385289 | ACTAATACCCATACAGTGGGGC | CTGAAGATCTCTTGCCAGCCTTC |
| **Acsm3** | 99538 | TCTGCGAACAGGGACAGTTT | CTGCTGGGGCCAAAGTATCA |
| **Acsm4** | 2681844 | ACTTAGATTCATCTGGCTTGCC | GGTAATGGCTTCTCACAGCGA |
| **Acsm5** | 2444086 | TATGCGAACAGGTGTGGTCA | TTTGGAGGGAGGGACAGTCA |
| **Acsl1** | 102797 | ATCTGGTGGAACGAGGCAAG | TCCTTTGGGGTTGCCTGTAG |
| **Acsl3** | 1921455 | TGTCTTTCTCATGGATGCCGA | CAGCACGGATGTGTCTCCTT |
| **Acsl4** | 1354713 | GCTATGACGCCCCTCTTTGT | GAATCGGTGTGTCTGAGGGG |
| **Acsl5** | 1919129 | CAGTGGAACTACAGGTGACCC | TACCCTGGACAAGCCTCTCA |
| **Acsl6** | 894291 | CCACAAGGCAACACTTCTGC | GTCCTCAAACGGCTCCATGA |
| **Fatp1** | 1347098 | TTCTCGTGGGCCAGATCAAC | AGCACGTCACCTGAGAGGTA |
| **Fatp2** | 1347099 | ATGATCGGCCTTCACGGATG | CCCGGTCATTTGGTTTCTGC |
| **Fatp3** | 1347358 | CTTCTGGTCTGGGGACGTTT | CCTTCCACCTGAAGGTGTCT |
| **Fatp4** | 1347347 | ACCCTACAAGGACGAGGAGT | AAGTAGCATTGTGACCCGGC |
| **Fatp5** | 1347100 | GCTGCTTACAAGTTGGAGCC | TGGTTGCTCAGGGACGTTAC |
| **Fatp6** | 3036230 | GTGCGTGTGCCAGGTTATGA | AAAACAGTGGGCAGGCGTAA |
| **Acsbg1** | 2385656 | ACTCGCAAACCAGCTCCTTA | CCGGGTTGTCCATAGTGCTT |
| **Acsbg2** | 3587728 | AGTCTCTGATCAAGCTCGGC | GATACTCACGTTGGCCTGCT |
| **Acsf1/Aacs** | 1926144 | CGTGTGGTCGGCTATCTACC | GCGGTCCAGGACACCATTTA |
| **Acsf2** | 2388287 | ATCTCAGTGGATGCCCCTCT | GTTGTCCCCGAGGTGAACTG |
| **Acsf3** | 2182591 | TTCAGAACAGGAGACACCGC | GGGCGCTGACTTTATAGCCT |
| **Acsf4** | 2442517 | GCCGTGAAACATGAATCCCC | ATACTTGGCCAGTGCCTTCG |
| **Acot1** | 1349396 | AACATCACCTTTGGAGGGGAG | TCCCCAACCTCCAAACCATCA |
| **Acot2** | 2159605 | AGTCAACGACGCAAAATGGTG | GCTCTTCCAATCCTGTTGGC |
| **Acot3** | 2159619 | GCTCAGTCACCCTCAGGTAA | AAGTTTCCGCCGATGTTGGA |
| **Acot4** | 2159621 | ACATCCAAAGGTAAAAGGCCCA | TCCACTGAATGCAGAGCCATT |
| **Acot5** | 2384969 | ACCCTCAGGTCAAGGGTCCA | TGAAATAACGTTGACCGTGGCG |
| **Acot6** | 1921287 | ATCCTCAGGTGAAAGGCCCAA | AAGGACAGTGGCTGTGATGTT |
| **Acot7** | 1917275 | ATCAGCACGCGGCACTGTAA | TTGGTACCTGTGAGGATGTTCTCC |
| **Acot8** | 2158201 | AAGTATCGAGTGGGGCTGAAC | TGATGTCACCTTCCCCAATGT |
| **Acot9/10** | 1928939/1928940 | GGGGCTTCTTACTCATGGCA | CATGGTCTCTCCAGACTGTGG |
| **Acot11** | 1913736 | GTGACCAGCGGCCCTTTAG | AGAACATAGAGGCGAAGCCCCTT |
| **Acot12** | 1921406 | CCGTGGCACTAAGGTCAGTT | ACGTTACGGTGCACGAATTG |
| **Acot13** | 1914084 | AGACTCTTGCTTTGCGTCCA | GACAAGCGTCACCTTTTCCAA |
| **Them4** | 1923028 | CAAAGGCCCAACAGTTCACC | ACCTCCGTGAACAAATCCAGG |
| **Them5** | 1913448 | CAGAGTCCCTGGTTGCAAGAT | CAGCCGCCTGATTTGGTTTT |
| **Rpl22** | 99262 | AGCAGGTTTTGAAGTTCACCC | CAGCTTTCCCATTCACCTTGA |
| **18S** | 97943 | GCAATTATTCCCCATGAACG | GGCCTCACTAAACCATCCAA |
| **Gapdh** | 95640 | AGGTCGGTGTGAACGGATTTG | TGTAGACCATGTAGTTGAGGTCA |
| **bactin** | 87904 | GGCTGTATTCCCCTCCATCG | CCAGTTGGTAACAATGCCATGT |
| **G6pase** | 95607 | CGACTCGCTATCTCCAAGTGA | GTTGAACCAGTCTCCGACCA |
| **Pepck** | 97501 | CTGCATAACGGTCTGGACTTC | CAGCAACTGCCCGTACTCC |
| **Pgc1a** | 1342774 | CAACATGCTCAAGCCAAACCAACA | CGCTCAATAGTCTTGTTCTCAAATGGG |
| **Ucp1** | 98894 | GTGAAGGTCAGAATGCAAGC | AGGGCCCCCTTCATGAGGTC |
